# An apricot story: view through a keyhole

**DOI:** 10.1186/1749-7922-2-20

**Published:** 2007-08-15

**Authors:** Tushar Samdani, Tarun Singhal, Santosh Balakrishnan, Abdulzahra Hussain, Starlene Grandy-Smith, Shamsi El-Hasani

**Affiliations:** 1 Department of General Surgery, Princess Royal University Hospital, Orpington, Kent, BR6 8ND, UK

## Abstract

**Background:**

Very few cases of small bowel obstruction due to ingested fruits have been described in literature, and most of these have managed by a laparotomy. Laparoscopic assisted surgery can effectively deal with such impacted foreign bodies, thereby avoiding a formal laparotomy.

**Case presentation:**

A 75 years old lady was admitted via the Accident and Emergency to the surgical ward with a three-day history of abdominal pain and vomiting. Investigations were suggestive of acute small bowel obstruction. On laparoscopy, there was an area of sudden change in calibre of small bowel with dilated proximal and collapsed distal segment in distal jejunum. A foreign body, dried undigested apricot, was extracted by mini-laparotomy.

**Discussion:**

Small bowel obstruction is a frequent cause of emergency surgery, and aetiology may include food bolus obstruction. Diagnosis is usually confirmed intra-operatively. Foreign body impacted in small bowel can be removed by open or laparoscopic methods.

**Conclusion:**

Generally, laparotomy is performed for diagnosis and management in acute bowel obstruction, but with increasing expertise, laparoscopy can be equally effective with all the other advantages of minimal access approach.

## Background

Foreign bodies are a known cause of bowel obstruction, especially so in children and the elderly. They present a diagnostic challenge because of the lack of history and the inability of the patient to correlate preceding events with the episode of bowel obstruction. Radiological investigations while helpful may not be diagnostic. Laparoscopy and laparoscopic assisted surgery can help resolve the problem with minimal trauma; and may help avoid a formal laparotomy in the unfortunate group of patients.

We would like to discuss the role of minimal access surgery in dealing with an intriguing case of small bowel obstruction due to an ingested apricot!

## Case presentation

A 75 years old lady was admitted via the Accident and Emergency to the surgical ward with a three-day history of abdominal pain and vomiting. The pain had started suddenly in the evening three days ago accompanied with vomiting three to four times. The vomitus consisted of dark brown fluid. The pain was initially in the left hypochondrial region and had lately become more generalised, constant, and increased in intensity. Her General Practioner, who had prescribed antiemetics for her symptoms, initially saw the patient. She had appendicectomy in the past, and had been diagnosed with diverticular disease on Barium enema and colonoscopy.

On examination, her vital parameters were normal. She had abdominal distension with generalised tenderness, abdomen was resonant on percussion, and she had decreased bowel sounds. Her routine blood tests were normal, and urine analysis showed increased ketones.

**Figure 1:**
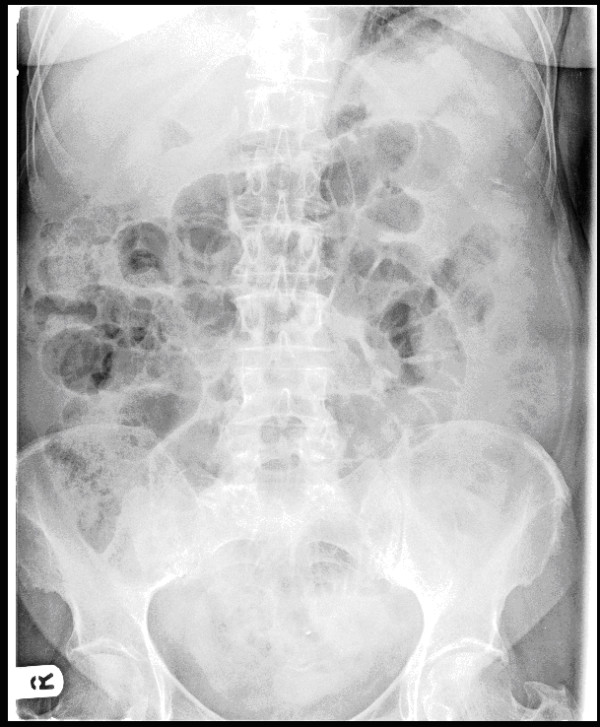
Abdominal X-Ray on the day of A&E admission showing small bowel dilation.

**Figure 2:**
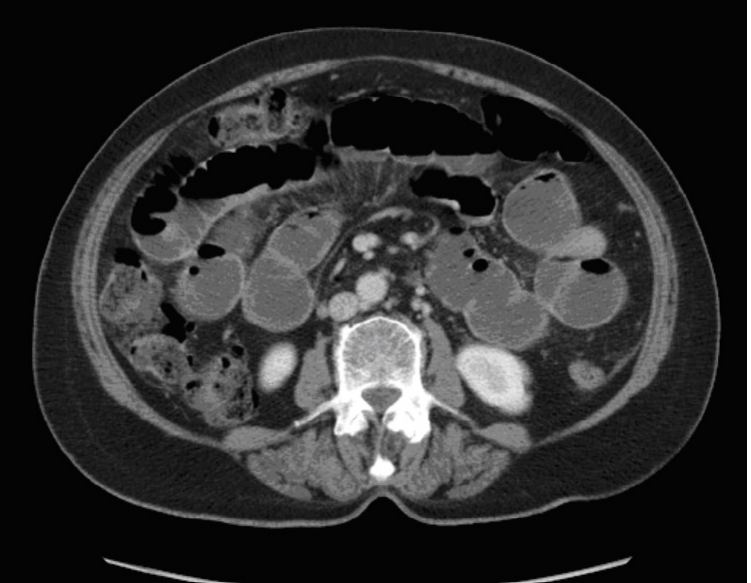
Emergency CT scan showing small bowel dilation with a point of abrupt change in calibre and angulation in the distal jejunal loop. The small bowel distal to this point was collapsed.

X-rays of the abdomen and chest were suggestive of dilated loops of small bowel (figure 1). A CT scan of the abdomen and pelvis showed dilated fluid filled small bowel loops with a point of abrupt change in calibre and angulation of bowel loop in the central abdomen. The small bowel beyond that point was collapsed. There was a small amount of free fluid in the pelvis. The CT scan was suggestive of adhesive small bowel obstruction, probably affecting a distal jejunal loop, with diverticular disease in sigmoid colon without any overt signs of active inflammation, and no free intraperitoneal air (figure 2).

**Figure 3:**
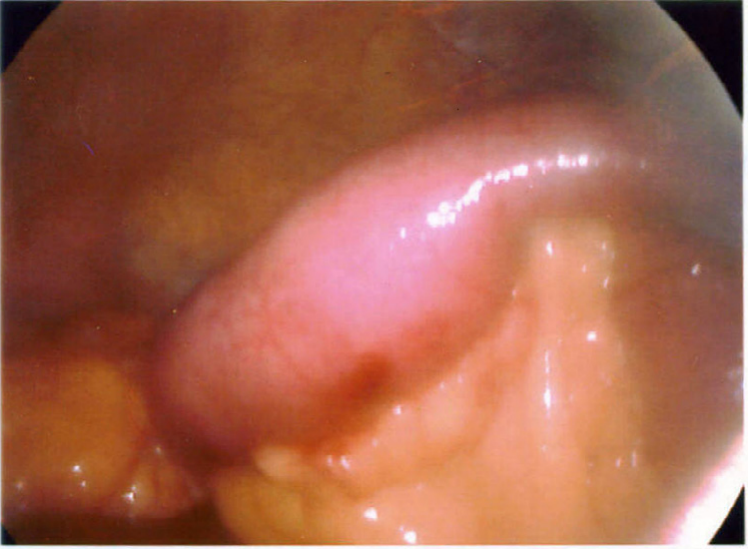
A view through laparoscope: showing dilatation of the distal jejunum with intraluminal body causing obstruction.

**Figure 4:**
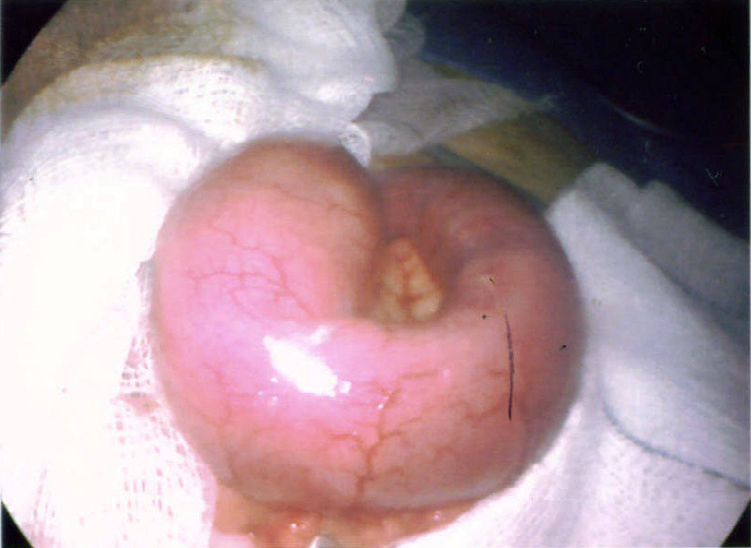
Dilated jejunum delivered through minilaparotomy.

**Figure 5:**
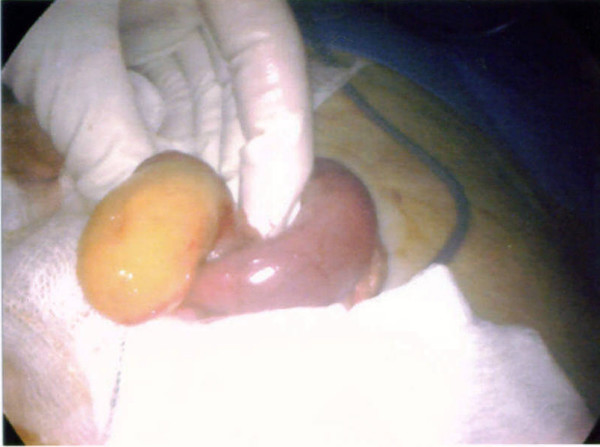
A foreign body, dried undigested apricot, was extracted through jejunum.

A clinical decision was made to proceed with laparoscopy. On laparoscopy, there was dilatation of the small intestine up to the distal jejunum (figure 3) with collapsed bowel beyond that point. The serosal surface of the small intestine looked normal. There were no adhesions between bowel loops; mesentery was normal with no evidence of lymphadenopathy. There was an area of sudden change in calibre with dilated proximal and collapsed distal segment in distal jejunum. The dilated segment of distal jejunum was brought out by a midline mini-laparotomy of five centimetres (figure 4). A foreign body, dried undigested apricot, was extracted (figure 5). The bowel at the point of impaction was normal and closed in two layers. Laparoscopically, we were unable to identify any other abnormality in the small bowel.

**Figure 6:**
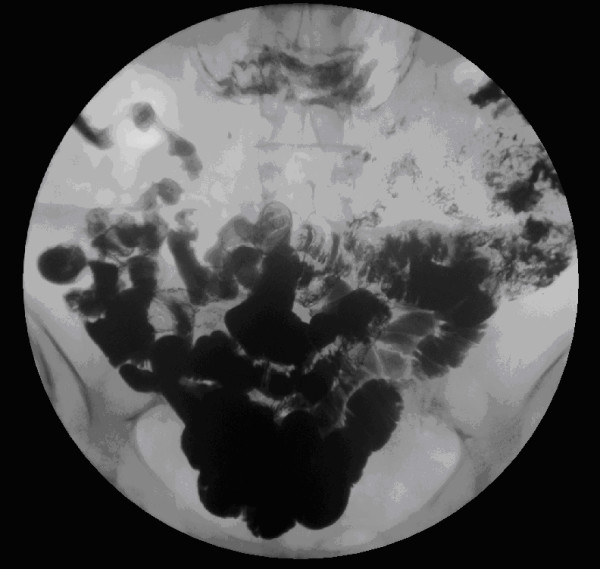
Post-operative Barium meal follow-through showing no pathology in the small intestine with passage of contrast into the colon. The contrast reached the terminal ileum after ninety minutes.

The patient had an uneventful post-operative recovery, and went home after ten days. On questioning she informed us that she had eaten few dried apricots while shopping three days before admission in A&E. She was followed up in the outpatient clinic with a Barium meal and follow-through, which was normal and did not show any abnormality in small intestine (figure 6).

## Discussion

Small bowel obstruction is a frequent cause of emergency surgery. Causes of small bowel obstruction are adhesion (60%), hernia (15%), neoplasm (6%), inflammatory (5%), and sometimes ingested foreign body, but rarely food bolus [1].

Patients with ingested foreign body are commonly children, elderly with dental prosthesis, alcoholic, prisoner inmates, and psychiatric patients [1-3]. Our patient did not fit into any of these categories. Coins, small toys, pins, and alkaline button batteries are some of the commonly ingested foreign bodies. Most of the ingested foreign bodies pass through the entire gastrointestinal tract without causing any complications, but if they are impacted in the gastro intestinal tract then they can cause obstruction, perforation, or fistula formation [1].

Food bolus impaction is common with meat, fish bones, and very few cases due to fruits have been reported [4]. It is seen in old people with poor natural teeth or ill-fitting dentures, or inadequate mastication as in our case [3]. Food bolus can get impacted at sites of narrowing in the gastro intestinal tract like cricopharyngeal sphincter, constriction in oesophagus (due to arch of aorta and bronchus), distal ileum (2 feet proximal to ileocaecal junction), ileocaecal junction and any pathological stricture in small bowel. Ingested foreign body longer than 6 cm is likely to be impacted in the second or third part of duodenum; however, rounded foreign bodies larger than 2.5 cm in diameter are less likely to pass beyond pylorus itself [2]. Presenting symptoms vary depending on site of impaction, type of ingested food, and presence or absence of complications. If food particle is impacted in oesophagus, then symptoms range from foreign body sensation, chest pain, odynophagia, vomiting, and respiratory symptoms.

Patients with impaction in small intestine present with symptoms of vomiting, abdominal distension, and constipation. There are cases of cholangitis [5] and recurrent pancreatitis [6] caused by food bolus impacted at papilla of Vater.

Sharp foreign bodies can perforate and present with mediastinitis or perforative peritonitis depending on site of perforation. Majority of the foreign bodies pass spontaneously and only 1% or less will require surgery [2].

Radiological investigations have limitations in studying bowel obstruction from foreign bodies, especially if when they are not radio-opaque. Plain abdominal film has sensitivity of 86% to diagnose high-grade bowel obstruction and will show air fluid level with dilated loops of small bowel [3,7]. An intramural width of small intestine of 3 cm is considered abnormal. Ultrasound may clearly demonstrate loops of distended small bowel with hyper peristalsis. Occasionally, the foreign body may be identified on ultrasound as an echogenic intraluminal mass and may cast an acoustic shadow if surrounded by fluid. When above investigations are inconclusive an abdominal CT scan is of great help in the diagnosis and detecting aetiology in 73–95% of cases [3,8].

Most ingested foreign bodies that have passed pylorus pass through rest of the gastro-intestinal tract, within a mean of 4 days. Ingested blunt foreign body distal to stomach are monitored by weekly abdominal X-rays, and daily X-rays in case of sharp objects. Intervention is required if the blunt foreign body remains in same place for more than a week, and sharp object remains in same place for more than 3 days [2].

Foreign bodies in oesophagus or stomach can be successfully removed endoscopically. Urgent endoscopic intervention is required in case of sharp objects, disk battery or if there is risk of aspiration. Under no circumstances should a foreign object or food bolus impaction be allowed to remain in the oesophagus beyond 24 hours from presentation [2]. Patients in whom endoscopic retrieval has failed are often referred for surgical extraction. Foreign body impacted in small bowel can be removed by open or laparoscopic methods. Proximal bowel should be checked for any other ingested foreign body. Careful examination of bowel at the site of impaction should be done to rule out a pathological stricture.

Early diagnosis and therapeutic management has considerable importance. Obstruction of the bowel due to impacted food bolus is difficult to diagnose preoperatively unless there is clear history and the diagnosis is usually made intraoperatively [4]. Generally, laparotomy is performed for diagnosis and management in such cases, but with increasing expertise, laparoscopy can be equally effective with all the other advantages of minimal access approach [9]. We recommend this approach for removal of ingested foreign body impacted in small intestine. The only disadvantage could be inability to feel the dilated bowel and conclusively rule out any other foreign body in proximal segment.
